# CDK activity at the centrosome regulates the cell cycle

**DOI:** 10.1016/j.celrep.2024.114066

**Published:** 2024-04-04

**Authors:** Emma L. Roberts, Jessica Greenwood, Nitin Kapadia, Tania Auchynnikava, Souradeep Basu, Paul Nurse

**Affiliations:** 1 Cell Cycle Laboratory, The Francis Crick Institute, NW1 1AT London, UK; 2 Protein Analysis and Proteomics Platform, The Francis Crick Institute, NW1 1AT London, UK; 3 Laboratory of Yeast Genetics and Cell Biology, Rockefeller University, New York, NY 10065, USA

**Keywords:** cell cycle, centrosome, cyclin B, CDK, mitosis, hydrophobic patch

## Abstract

In human cells and yeast, an intact “hydrophobic patch” substrate docking site is needed for mitotic cyclin centrosomal localization. A hydrophobic patch mutant (HPM) of the fission yeast mitotic cyclin Cdc13 cannot enter mitosis, but whether this is due to defective centrosomal localization or defective cyclin-substrate docking more widely is unknown. Here, we show that artificially restoring Cdc13-HPM centrosomal localization promotes mitotic entry and increases CDK (cyclin-dependent kinase) substrate phosphorylation at the centrosome and in the cytoplasm. We also show that the S-phase B-cyclin hydrophobic patch is required for centrosomal localization but not for S phase. We propose that the hydrophobic patch is essential for mitosis due to its requirement for the local concentration of cyclin-CDK with CDK substrates and regulators at the centrosome. Our findings emphasize the central importance of the centrosome as a hub coordinating cell-cycle control and explain why the cyclin hydrophobic patch is essential for mitosis.

## Highlights

• Cyclin B centrosomal localization is needed for mitotic entry in fission yeast
• Centrosomal cyclin-CDK promotes substrate phosphorylation at the centrosome and cytoplasm
• The same substrate docking region targets both S- and M-phase B-cyclin to the centrosome
• S-phase cyclin localization to the centrosome is not necessary for DNA replication

## Graphical abstract

**Figure:**
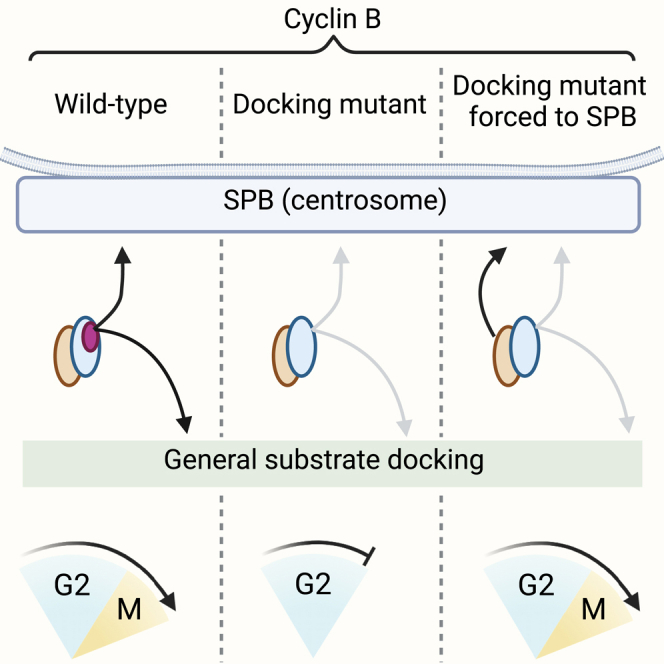

## Teaser

Roberts et al. find that yeast cyclin B-CDK centrosomal localization is essential for mitotic entry and promotes phosphorylation of centrosomal and cytoplasmic CDK substrates. The same hydrophobic patch substrate docking region mediates the localization of both S- and M-phase B-cyclin to the centrosome and is not required for S phase.

## Introduction

The conserved hydrophobic patch (HP) docking site on cyclins binds to cyclin-dependent kinase (CDK) substrates and activity regulators^1^^,^^2^^,^^3^^,^^4^^,^^5^^,^^6^ and mediates localization of cyclins including human cyclin B1 and the B-cyclin Cdc13 in the fission yeast *Schizosaccharomyces pombe* to the centrosome and spindle pole body (SPB; yeast centrosome equivalent).^7^^,^^8^^,^^9^ Although the HP is a critical point of CDK activity regulation, with the Cdc13 HP being essential for mitosis,^7^ it is unclear why.

In fission yeast, signaling based around the SPB component Cut12 can influence mitotic entry through CDK and the mitotic regulator Plo1 (Plk1 in humans).^10^^,^^11^^,^^12^^,^^13^ Artificial SPB recruitment of Cdc2-Y15F (which is insensitive to the inhibitory kinase Wee1) is sufficient to promote mitotic entry in G2 cells.^14^ Furthermore, signaling at the centrosome in the *C. elegans* early embryo can influence the timing of nuclear envelope breakdown,^15^^,^^16^ suggesting that centrosomes may also act as a signaling hub regulating mitotic entry. Supporting this, during mitosis, an active form of cyclin B1-Cdk1 is first detected at the centrosomes in HeLa cells.^17^ However, centrosomes are not required for cell division in *Drosophila* after the early embryo stage,^18^ and removal of centrosomes in some cultured mammalian cell lines does not prevent mitotic entry or progression.^19^^,^^20^^,^^21^^,^^22^ Therefore, although cyclin-CDK has been seen to localize to the centrosome in many eukaryotic species, questions remain around how important this conserved phenomenon is for cell-cycle regulation.

In addition to mediating the localization of cyclin-CDK, the HP docks to a number of CDK substrates and regulators, enhancing substrate phosphorylation or influencing CDK activity regulation, and these are disrupted upon mutating the HP.^1^^,^^2^^,^^3^^,^^4^^,^^5^ Thus, it remains unknown whether the essential *in vivo* function of the Cdc13 HP lies in its role in cyclin-CDK SPB localization specifically or in its wider role in substrate docking. Furthermore, how conserved the different roles of the HP are between S- and M-phase cyclins, and the *in vivo* importance of these roles, remains unclear. The HP of budding yeast S- and M-phase cyclins enhances *in vitro* phosphorylation of different substrates^3^ and can recognize different docking motifs on substrates,^5^^,^^6^ indicating some differences in the HP of different cyclins. However, the HPs of both mammalian cyclins A2 and B1 mediate their centrosomal localization.^7^^,^^8^^,^^9^

Here, we investigate the importance of the HP and centrosomal cyclin-CDK localization in S- and M-phase cyclins *in vivo* using the relatively simple *S. pombe* cell cycle. We artificially restored the SPB localization of a HP mutant (HPM), Cdc13-HPM, and found that this promotes mitotic entry and increases CDK substrate phosphorylation at both the SPB and in the cytoplasm. We also found that the HP of the S-phase cyclin Cig2 is not essential for bulk DNA replication but is involved in SPB localization. Our results demonstrate that SPB localization of the mitotic cyclin Cdc13 is essential for mitotic entry, explaining why the HP of Cdc13 is essential for cell cycle progression.

## Results

### Cells driven by Cdc13-HPM arrest in G2 without Cdc13-HPM SPB localization

**Figure 1:**
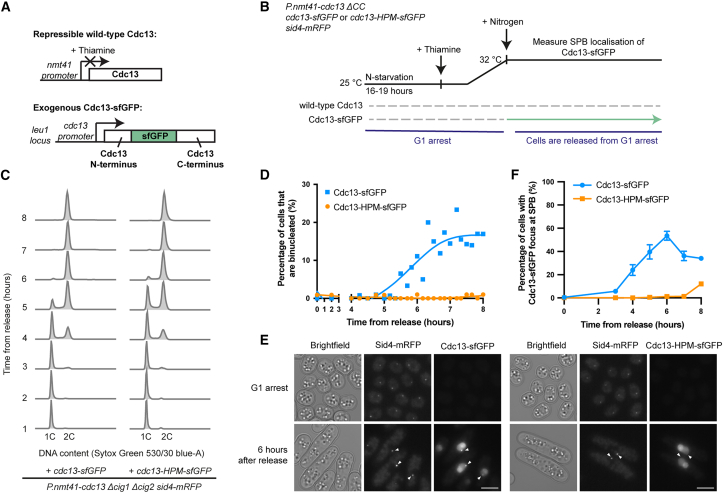
Cells driven by Cdc13-HPM arrest in G2 without detectable Cdc13-HPM SPB enrichment (A) Schematic of Cdc13 conditional expression system. The sfGFP tag is internal to Cdc13.^23^ (B) Experimental design. Nitrogen starvation results in a G1 arrest, during which Cdc13 protein is degraded. Cells were released from the arrest by nitrogen addition. (C) DNA content of cells after release from the G1 arrest determined by flow cytometry. (D) Heat-fixed cells were stained with DAPI and scored for binucleation to measure passage through mitosis. *n* > 70 cells per time point; spline was plotted in GraphPad Prism. (E) Cdc13-sfGFP localization in cells arrested in G1 and 6 h after release. Arrows indicate location of SPBs as judged by Sid4-mRFP signal. Scale bars, 5 μm. (F) Cells were scored for Cdc13-sfGFP and Cdc13-HPM-sfGFP SPB localization. Mean and SD of two biological replicates are shown except for time points 3 (wild type) and 8 (wild type and HPM), where there are data from one replicate. *n* > 240 cells per strain per time point. See also Figure S1.

We previously found that in the presence of endogenous wild-type Cdc13, an exogenous copy of an HPM,^2^ Cdc13-HPM, is unable to localize to the SPB in early G2 but can localize to the SPB in late G2/mitosis.^7^ Thus, although cells expressing only Cdc13-HPM arrest in G2,^7^ it is unknown whether they arrest before Cdc13-HPM can localize to the SPB. To investigate this, we introduced into cells an exogenous copy of either Cdc13-sfGFP (superfolder GFP)^23^ or Cdc13-HPM-sfGFP and placed wild-type Cdc13 under a thiamine-repressible promoter (Figure 1A). The G1/S cyclin genes *cig1* and *cig2* were deleted to simplify overall CDK cell-cycle control, and an SPB component, Sid4-mRFP,^24^ was used as an SPB marker. Cells were followed after release from a G1 arrest with wild-type *cdc13* repressed (Figure 1B). As expected,^7^ cells expressing Cdc13-sfGFP and Cdc13-HPM-sfGFP both performed S phase at similar times after release (Figure 1C). However, although cells expressing Cdc13-sfGFP underwent mitosis, forming binucleates, cells expressing Cdc13-HPM-sfGFP did not undergo mitosis (Figure 1D).

We determined whether Cdc13-HPM-sfGFP could localize to the SPB using an automated spot detection pipeline (Figure S1). The proportion of cells with Cdc13-sfGFP at the SPB increased significantly with a peak of roughly 50% at 6 h after release from G1 arrest and then decreased as cells underwent mitosis and degraded Cdc13 (Figures 1D–1F). In contrast, the proportion of cells with Cdc13-HPM-sfGFP at the SPB was essentially negligible (Figures 1E and 1F). We conclude that cells driven by Cdc13-HPM-sfGFP arrest in G2 without detectable Cdc13-HPM-sfGFP present at the SPB and that this SPB localization defect of Cdc13-HPM is a potential cause of its failure to undergo mitosis.

### Artificial restoration of Cdc2 SPB localization

The mitotic entry defect of Cdc13-HPM could be caused by its SPB localization defect, a disruption of cyclin-substrate docking more widely, or a combination of both. To test the importance of Cdc13 SPB localization, we tested whether artificially restoring Cdc13-HPM SPB localization rescued the mitotic defect of Cdc13-HPM. To do this, we adapted an experimental approach previously used to tether CDK to the SPB^14^ (Figures S2A–S2C). Cdc13-HPM was introduced into cells with thiamine-repressible wild-type *cdc13* (Figure S2A). An exogenous copy of the analog-sensitive allele^25^ Cdc2-asM17-GBP-mCherry (sensitive to inhibition by 1-NmPP1) was tethered to the SPB using a tagged SPB component, Cut12-NEGFP^26^^,^^27^ (Figure S2C). Cut12, a core SPB component important for SPB activation and integration into the nuclear envelope,^28^ was selected, as it is has previously been implicated in CDK regulation of mitosis.^11^^,^^27^^,^^29^ This includes the finding that tethering a constitutively active allele of Cdc2 to SPB components promotes mitotic entry, with the strongest phenotype observed when Cdc2 was tethered to Cut12.^14^

**Figure 2:**
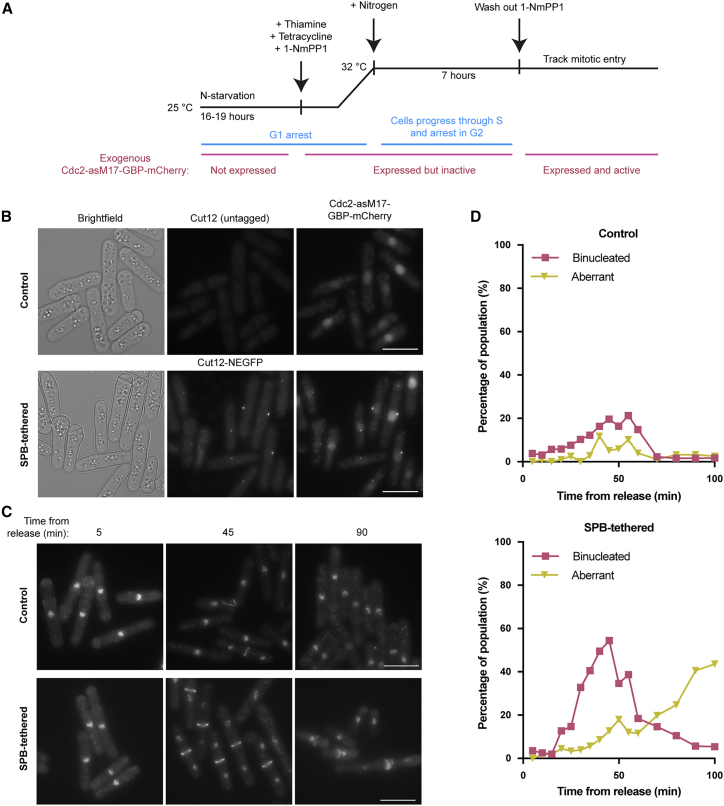
Artificially restoring Cdc13-HPM SPB localization promotes mitotic entry (A) Experimental outline. (B) Images of the indicated strains 6 h after release from the G1 arrest (7 h after tetracycline addition). Scale bar, 10 μm. (C) DAPI and calcofluor staining of the DNA and septum, respectively, taken at the indicated time after 1-NmPP1 washout. The displayed pixel range is the same for all images of the same strain but not between strains; pixel range is chosen to best represent DNA and septum staining. Scale bars, 10 μm. (D) Cells were heat-fixed at the indicated time points after 1-NmPP1 washout and stained with DAPI and calcofluor to score for passage through mitosis. “Aberrant” includes multiseptate and cut cells. *n* ≥ 90 cells per time point. See also Figure S2.

We first arrested cells in G1, repressing wild-type *cdc13* expression and inducing Cdc2-asM17-GBP-mCherry expression in the presence of 1-NmPP1 to inhibit its CDK activity (Figures 2A and 2B). Thus, the only active copy of Cdc2 was endogenous Cdc2. Upon release from G1 arrest, cells performed S phase but arrested in G2, as expected for cells driven by Cdc13-HPM (Figures S2D and S2E). Once cells had accumulated in G2, 1-NmPP1 was removed from the cultures to activate Cdc2-asM17-GBP-mCherry, and cells were monitored for progression through mitosis (Figure 2A).

The SPB-tethered strain efficiently underwent mitosis with peaks of binucleates of 30%–50% in repeat experiments (Figures 2C, 2D, and S2F). In contrast, the non-tethered control strain underwent a much reduced level of mitosis with peaks of binucleates at 10%–20% (Figures 2C, 2D, and S2F). In the SPB-tethered strain, there was also an accumulation of aberrant nuclear divisions in later time points indicative of cells that had entered mitosis and encountered difficulties in mitotic progression (Figures 2C and 2D). This could be due to the artificial SPB tethering system failing to precisely recapitulate normal SPB localization or the lack of cyclin-substrate docking. We conclude that artificially restoring the localization of Cdc2 at the SPB significantly increases the number of cells entering and proceeding through mitosis and propose that mitotic entry is dependent upon Cdc13 localization to the SPB, which requires the HP.

### Artificial restoration of Cdc2 SPB localization and CDK substrate phosphorylation

**Figure 3:**
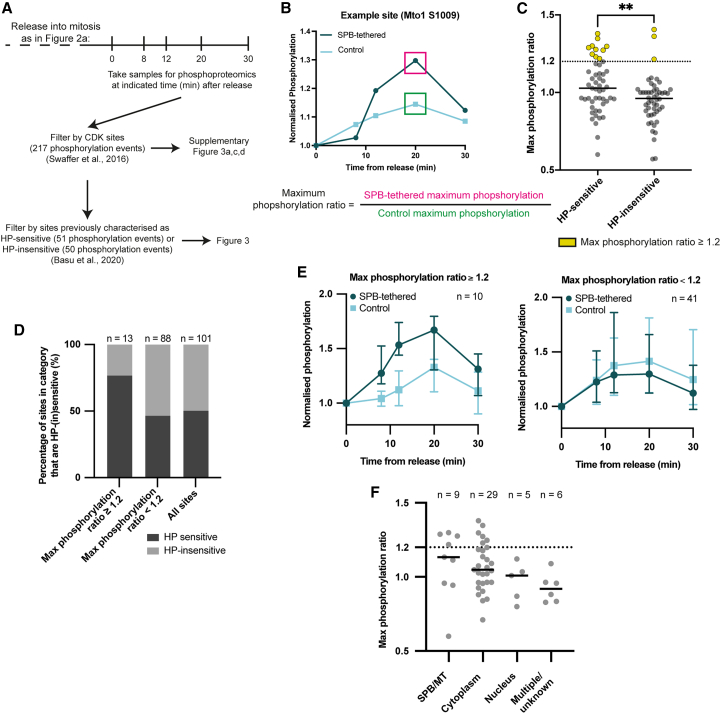
Artificially restoring Cdc13-HPM SPB localization partially rescues CDK substrate phosphorylation at the SPB and cytoplasm (A) Schematic of phosphoproteomics experiment and key steps in subsequent analysis. Previously published time course experiments from Swaffer et al.^30^ and Basu et al.^7^ are used to define CDK sites and HP sensitivity, respectively. (B) Schematic to show the calculation of maximum phosphorylation ratio of an example phosphosite. (C) Maximum phosphorylation ratio of HP-sensitive and HP-insensitive sites. Solid bars show median of the group. Mann-Whitney rank comparison performed to compare entire groups; ^∗∗^*p* = 0.0052. (D) The percentage of sites in each category that are HP sensitive or insensitive. (E) Median of the normalized phosphorylation of HP-sensitive sites separated according to their maximum phosphorylation ratio. Error bars, 95% confidence intervals. (F) The maximum phosphorylation ratio of HP-sensitive sites split according to their reported localization.^7^ Solid bars show median of the group. MT = microtubules. See also Figure S3 and Table S1.

Given that disrupting the HP of Cdc13 results in defective phosphorylation of substrates localized both at the SPB and within the cytoplasm,^7^ we tested if artificial restoration of Cdc2 SPB localization rescued substrate phosphorylation in both these subcellular compartments. We repeated the experiment in Figure 2 and took samples for quantitative phosphoproteomics after 1-NmPP1 removal (Figure 3A). We identified 217 previously defined CDK phosphorylation^30^ events in our dataset (Table S1). Of these, 51 phosphorylation events had been previously classified as HP sensitive (sites phosphorylated less by Cdc13-HPM than Cdc13) and 50 phosphorylation events as HP insensitive (sites phosphorylated to wild-type levels by Cdc13-HPM).^7^ We also confirmed that CDK substrate phosphorylation was comparable between both strains before 1-NmPP1 removal (Figure S3A).

To identify sites that increased in phosphorylation as a result of restoring Cdc2 SPB localization, we determined the ratio of the maximum phosphorylation reached by the SPB-tethered strain and the control strain for each HP-(in)sensitive site (Figure 3B). A maximum phosphorylation ratio at least 1.2 (indicating maximum phosphorylation in the SPB-tethered strain was at least 20% higher than the control strain) was used as a threshold to identify sites with increased phosphorylation, which were predominantly HP sensitive (Figures 3C and 3D).

Approximately one-fifth of HP-sensitive sites had a maximum phosphorylation ratio of at least 1.2, indicating some rescue of phosphorylation (Figure 3E). These included some, but not all, HP-sensitive sites from the SPB/MTs and the cytoplasm (Figures 3F and S3B). This suggests that tethering Cdc2-asM17-GBP-mCherry to Cut12-NEGFP promoted some rescue of CDK substrate phosphorylation both at the SPB and within the cytoplasm. We also extended our analysis to include CDK phosphorylation sites in key G2/M regulators and found that some had increased phosphorylation in the SPB-tethered strain (Figure S3C). These included a site on Plo1, which has previously been shown to interact with Cut12^11^ (Figure S3D).

We conclude that artificial tethering of Cdc2-Cdc13-HPM to the SPB increases phosphorylation of CDK substrates located both at the SPB and within the cytoplasm. These results support the view that the SPB is a critical hub for controlling CDK phosphorylation and mitosis.^13^^,^^21^

### Cig2-HPM can drive S phase but has reduced SPB localization

**Figure 4:**
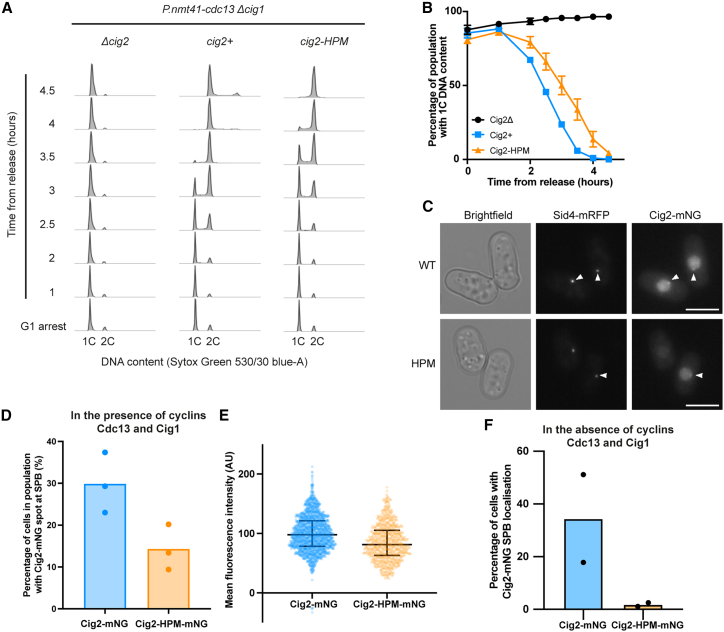
Cig2-HPM can drive DNA replication but is not detected at the SPB in the absence of other cyclins (A and B) Cells expressing *P.nmt41-cdc13 cig1Δ* and either *cig2+*, *cig2-HPM*, or no copy (*cig2Δ*) were followed after release from a G1 arrest. (A) DNA content of one of three replicates. (B) The percentage of cells with 1C DNA content; mean and SD of three replicates are shown except for the *cig2+* strain at 1 and 2 h, where there are two replicate values. (C–E) An asynchronous population of cells expressing either *cig2-mNG* or *cig2-HPM-mNG* as well as *sid4-mRFP* to mark the SPB was imaged. (C) Cells with Cig2-mNG and Cig2-HPM-mNG at the SPB (marked by arrowheads). Scale bars, 5 μm. (D) Percentage of cells in the population with Cig2-mNG and Cig2-HPM-mNG at the SPB; bar shows median of three replicates. (E) Mean fluorescence intensity of Cig2-mNG and Cig2-HPM-mNG; dots represent individual cells and bars represent the population median and IQR. One replicate of three is shown. Cig2-mNG *n* = 1,658 and Cig2-HPM-mNG *n* = 746 cells. (F) Cells with *cdc13* repressed, *cig1Δ*, *sid4-mRFP*, and either *cig2-mNG* or *cig2-HPM-mNG* were released from a G1 arrest and followed with time-lapse imaging. Graph shows percentage of cells with Cig2-mNG at the SPB at any point during the time-lapse. Bar shows median of two replicates. See also Figure S4 and Videos S1 and S2.

Our results indicate that mediating centrosomal localization is a major role of the HP in mitotic cyclins. However, M- and S-phase cyclins have been shown to recognize different motifs in substrates,^5^^,^^6^ and cyclin-substrate docking is thought to be more widespread for S-phase cyclins,^3^ indicating that there may be some differences in the HPs of S- and M-phase cyclins. We therefore asked if the involvement of the HP in centrosomal localization, and its importance in cyclin function, is conserved in the S-phase cyclin Cig2. The budding yeast S-phase cyclin HP enhances *in vitro* phosphorylation of early substrates involved in DNA replication,^3^ and so we tested if Cig2-HPM (M168A, L172A, W175A) is able to drive DNA replication *in vivo*. We released *P.nmt41-cdc13 cig1Δ* cells from a G1 arrest with *cdc13* expression repressed and with *cig2+*, *cig2-HPM*, or *cig2Δ* at the endogenous locus and followed S-phase progression using flow cytometry. The populations expressing Cig2 and Cig2-HPM both underwent bulk DNA replication, albeit with a delay in cells with Cig2-HPM compared to Cig2 (Figures 4A and 4B). We conclude that an intact HP is not essential *in vivo* for Cig2 to drive S phase.

We also investigated Cig2-HPM SPB localization. Endogenous Cig2-mNeonGreen (mNG) and Cig2-HPM-mNG were both detected at the SPB in relatively short cells, indicating that they were early in the cell cycle, which suggest that, like Cdc13,^7^ there is an HP-independent mechanism of Cig2-mNG SPB localization (Figures 4C, 4D, and S4A).

Roughly twice as many cells had detectable Cig2-mNG SPB foci than Cig2-HPM-mNG (Figure 4D). This indicates that as with Cdc13, the HP of Cig2 is also involved in its localization to the SPB. However, Cig2-HPM-mNG is expressed at a lower level in the population, although it is still 80% of the Cig2-mNG level (Figure 4E), and this may contribute to a reduction in detectable Cig2-HPM-mNG SPB foci.

We further introduced the Y136A mutation, which is equivalent to the mutation previously shown to disrupt cyclin B1-CDK interaction.^8^ This reduced detectable Cig2-mNG SPB localization (Figure S4B). There was virtually no Cig2-HPM-Y136A-mNG SPB localization detected, suggesting that the HP-independent SPB localization of Cig2 requires CDK association (Figure S4B). The HP-independent mechanism of Cdc13 SPB localization is not detected in the absence of wild-type Cdc13, Cig1, and Cig2 (Figures 1E and 1F), prompting us to ask whether the same was true for Cig2. We released *Δcig1* cells from a G1 arrest while repressing *cdc13* transcription and monitored endogenous Cig2-mNG localization by time-lapse microscopy. We altered our spot detection workflow (Figure S1) to take account of the Sid4-mRFP signal being too weak to mask the SPB in all time points (see STAR Methods). In contrast to Cig2-mNG, there were almost no cells with Cig2-HPM-mNG SPB localization (Figures 4F and S4C; Videos S1 and S2). The decrease in detectable SPB foci was not due to the lower expression of Cig2-HPM-mNG, as restricting the analysis to cells of similar expression levels did not affect the result (Figures S4D and S4E). Thus, we found that in the absence of Cdc13 and Cig1, no Cig2-HPM-mNG enrichment was detected at the SPB. This suggests that the HP of Cig2 mediates its localization to the SPB, and there is an HP-independent mechanism of Cig2-mNG SPB localization that requires CDK association and does not occur in the absence of wild-type Cig2, Cdc13, and Cig1. We conclude that the role of the HP in mediating SPB localization is conserved between S- and M-phase cyclins but, like Cdc13,^7^ this localization is not needed for Cig2 to drive bulk DNA replication.

Video S1. Cig2-mNG localization after release from G1 arrest, related to Figures 4 and S4 and Video S2

Video S2. Cig2-HPM-mNG localization after release from G1 arrest, see also Figures 4 and S4 and Video S1

## Discussion

Despite evidence in multiple eukaryotes implicating signaling at the centrosome in cell-cycle regulation,^10^^,^^11^^,^^12^^,^^13^^,^^14^^,^^15^^,^^16^^,^^17^^,^^31^ the role of cyclin-CDK centrosomal localization remains an open question. We have shown here using fission yeast that cyclin-CDK SPB localization is essential for mitotic entry. The blockage of mitosis caused by mutating the mitotic cyclin Cdc13 HP can be largely reversed by artificially tethering Cdc2 to the centrosome equivalent (SPB). This establishes that mitotic cyclin localization at the centrosome plays a critical role in mitotic regulation.

As well as promoting mitotic entry, artificially restoring Cdc13-HPM localization at the SPB rescues CDK phosphorylation of some, but not all, CDK substrates whose phosphorylation is reduced by mutating the HP. We therefore propose that the HP enhances mitotic CDK substrate phosphorylation partly through centrosomal localization, which is likely mediated by docking to SPB/centrosomal component(s) that may themselves be CDK substrates. The observation that not all phosphorylation sites are rescued suggests either that the artificial tethering does not fully recapitulate the normal centrosomal localization or that phosphorylation of some substrates requires their direct docking via the HP. We favor the latter explanation given the extensive evidence that the HP acts as a substrate docking site.^1^^,^^2^^,^^3^^,^^4^^,^^5^^,^^6^^,^^32^

The proteins that Cdc13 docks to at the SPB with the HP are unknown but could include Sad1, Ppc89, and/or Cdc11, which are reported to interact with Cdc13.^33^^,^^34^ Given that both Cig2 and Cdc13 dock to the SPB via the HP, they may interact with the same protein(s) there. However, differences in the HPs of Cig2 and Cdc13 could result in their interaction with different SPB proteins.

Our work supports the evidence in both yeast^10^^,^^11^^,^^12^^,^^13^^,^^14^^,^^31^ and metazoa^15^^,^^16^^,^^17^ implicating the centrosome as a signaling hub^21^ and extends it by demonstrating that cyclin-CDK localization to the fission yeast SPB is essential for mitosis. Co-localization of the mitotic cyclin-CDK and mitotic substrates at the centrosome could increase the local concentrations of CDK and its substrates, resulting in more efficient phosphorylation. In addition, the concentration of CDK regulators at the centrosome could promote positive feedback of CDK activity.^10^^,^^13^^,^^14^^,^^21^ However, why is it that *Drosophila* cells and some vertebrate cell lines can undergo mitosis without a centrosome^18^^,^^19^^,^^20^^,^^21^? One possible explanation is that it is not concentration at the centrosome that is essential but rather concentration per se.^22^ If there is no centrosome, concentration at another location may be able to substitute for the centrosome.^22^

We also found that CDK substrates whose phosphorylation was increased by artificially locating cyclin Cdc13-HPM to the centrosome were localized in the cytoplasm as well as the centrosome. Increased phosphorylation of centrosomal CDK substrates is to be expected given the tethering of CDK to that location. The increase observed for cytoplasmic CDK substrates could be explained by different mechanisms. For example, CDK could be activated at the centrosome and this activity propagated to the cytoplasm,^13^^,^^14^ or CDK substrates could be phosphorylated at the centrosome and then diffuse into the cytoplasm. Both mechanisms implicate the centrosome as a significant mitotic regulator.

Finally, we have shown that the HP in fission yeast S-phase cyclin Cig2 mediates its localization to the SPB centrosome as it does for the mitotic cyclin Cdc13^7^ and for the mammalian cyclins A2 and B1.^7^^,^^8^^,^^9^ In addition, we found that an intact HP of Cig2 is not required for S phase. This is despite the observations that the HP of the budding yeast S-phase cyclin enhances phosphorylation of S-phase substrates *in vitro* and that the centrosomal localization of mammalian cyclins A and E promotes DNA replication.^9^^,^^35^

Overall, our results emphasize the central importance of the centrosomal localizations of cyclin-CDK for mitotic regulation. The localizations of a number of core cell-cycle regulators, including cyclin-CDK at the metazoan centrosome,^21^^,^^36^^,^^37^^,^^38^ and the conservation of the role of the cyclin B HP in centrosomal localization^7^^,^^8^ suggest that our findings are also relevant for mitotic control more generally throughout eukaryotes.

### Limitations of the study

Due to the experimental system required to synchronize the cells in Figures 2 and 3 in G2, a strain expressing wild-type Cdc13 cannot be used to identify wild-type Cdc13-CDK substrate phosphorylation levels in this genetic background. Additionally, although conclusions can be drawn regarding sites that have detectable differences in phosphorylation between the SPB-tethered and control strains, there are several reasons explaining why some sites have the same phosphorylation in both strains. These sites could rely on direct docking to the HP, their phosphorylation could be rescued by the overexpression of Cdc13 and Cdc2 in the experiment, or the phosphorylation increase upon rescue could be too small to distinguish in this experiment.

## STAR★Methods

### Key resources table

**Table:** 

REAGENT or RESOURCE	SOURCE	IDENTIFIER
**Chemicals, peptides, and recombinant proteins**		

Thiamine Hydrochloride	Sigma Aldrich	Cat# T4625
cOmplete mini protease inhibitor cocktail	CalBiochem	Cat# 11836153001
PhosSTOP phosphatase inhibitor tablets	Roche	Cat# PHOSS-RO
Anhydrotetracycline hydrochloride	Sigma Aldrich	Cat# 37919
Sytox Green	Invitrogen	Cat# S7020
1-NmPP1	Toronto Research Chemicals	Cat# A603003
SlowFade Diamond Antifade Mountant with DAPI	Invitrogen	Cat# S36968
Calcofluor	Sigma Aldrich	Cat# 18909
Pierce Trypsin Protease, MS Grade	ThermoFisher	Cat# 90058

**Critical commercial assays**		

Dynabeads Protein A	ThermoFisher	Cat# 10002D
Dynabeads M-270 Epoxy	ThermoFisher	Cat# 14302D
TMT 10-plex Isobaric Label Reagent Set	ThermoFisher	Cat# 90110
Pierce TiO2 Phosphopeptide Enrichment Spin Kits	ThermoFisher	Cat# 88303
High-Select Fe-NTA Phosphopeptide enrichment kit	ThermoFisher	Cat# A32992
Pierce High pH Reverse-Phase peptide Fractionation kit	ThermoFisher	Cat# 84868
Gene frame	ThermoFisher	Cat# AB-0577
UltiMate 3000 HPLC System	ThermoFisher	Cat# 5041.0010
EASY-Spray C18 Column, 75 mm × 50 cm	ThermoFisher	Cat# ES803
Orbitrap Eclipse Tribrid Mass Spectrometer	ThermoFisher	Cat# FSN04-10000

**Deposited data**		

The mass spectrometry proteomics data obtained in This manuscript have been deposited to the ProteomeXchange Consortium via the PRIDE partner repository.	This manuscript	PRIDE: PXD044732
Original code has been uploaded to GitHub and can be accessed via Zenodo	This manuscript	https://doi.org/10.5281/zenodo.10371062

**Experimental models: Organisms/strains**		

S. pombe: IH1356: h- cut12-NEGFP:ura4+ ura4-D18 leu1-32	Bridge et al.^27^	IH1356
S. pombe: ST946: h90 cdc13-sfGFP	Kamenz et al.^23^	ST946
S. pombe: PN5478: h+ cdc2-asM17:bsdMX6	Aoi et al.^25^	PN5478
S. pombe: PN1813: h+ P.nmt41-cdc13+:LEU2+ cdc13Δ:ura4+ cig1Δ:ura4+ cig2Δ:ura4+ ura4-D18 ade6-M210 leu1-32	Nurse lab stock	PN1813
S. pombe: PN6011: h+ cdc13Δ:ura4+ P.nmt41-cdc13+:LEU2+ leu1Δ::P.cdc13-cdc13-M235A,L239A,W242A-sfGFP-T.cdc13:hphMX6 Δcig1:ura4+ Δcig2:ura4+ sid4-mRFP:kanMX6 ade6-M210 ura4-D18	This manuscript	PN6011
S. pombe: PN6012: h+ cdc13Δ:ura4+ P.nmt41-cdc13+:LEU2+ leu1Δ::P.cdc13-cdc13-sfGFP-T.cdc13:hphMX6 Δcig1:ura4+ Δcig2:ura4+ sid4-mRFP:kanMX6 ade6-M210 ura4-D18	This manuscript	PN6012
S. pombe: PN6033: h- cdc13Δ:ura4+ P.nmt41-cdc13+:LEU2+ cig1Δ:ura4+ cig2Δ:ura4+ ura4-D18 pDM291-tetR-tup11Δ70:ura4+ cut12-NEGFP:ura4+ leu1Δ::P.cdc13-cdc13-M235A,L239A,W242A-T.cdc13:hphMX6 lys1+:P.tet-cdc2-asM17-GBP-mCherry-T.adh1:natMX6 ade6-M210	This manuscript	PN6033
S. pombe: PN6034: h+ cdc13Δ:ura4+ ura4-D18 P.nmt41-cdc13+:LEU2+ cig1Δ:ura4+ cig2Δ:ura4+ pDM291-tetR-tup11Δ70:ura4+ leu1Δ: P.cdc13-cdc13-M235A,L239A,W242A-T.cdc13:hphMX6 lys1+:P.tet-cdc2-asM17-GBP-mCherry-T.adh1:natMX6 ade6-M210	This manuscript	PN6034
S. pombe: PN5740: h+ sid4-mRFP:kanMX6 ura4-D18 ade6- leu1-32	Kume et al.^24^	PN5740
S. pombe: PN6042: h+ cig2-M168A,L172A,W175A-mNeonGreen:kanMX6 sid4-mRFP:kanMX6 ade6-M216 leu1-32 ura4-D18	This manuscript	PN6042
S. pombe: PN6044: h+ cig2-mNeonGreen:kanMX6 sid4-mRFP:KanMX6 ade6-M216 leu1-32 ura4-D18	This manuscript	PN6044
S. pombe: PN6045: h- cdc13Δ:ura4+ P.nmt41-cdc13+:LEU2+ Δcig1:ura4+ cig2-M168A,L172A,W175A-mNeonGreen:kanMX6 sid4-mRFP:kanMX6 ade6-	This manuscript	PN6045
S. pombe: PN6046: h- cdc13Δ:ura4+ P.nmt41-cdc13:LEU2+ Δcig1:ura4+ cig2-mNeonGreen:kanMX6 sid4-mRFP:kanMX6 ade6-	This manuscript	PN6046
S. pombe: PN6039: h+ cdc13Δ:ura4+ ura4-D18(?) P.nmt41-cdc13:LEU2+ leu1-32(?) ade6-M210 cig1Δ:ura4+ cig2:natMX6	This manuscript	PN6039
S. pombe: PN6040: h- cdc13Δ:ura4+ ura4-D18(?) P.nmt41-cdc13:LEU2+ leu1-32(?) ade6-M210 cig1Δ:ura4+ cig2-M168A,L172A,W175A:natMX6	This manuscript	PN6040
S. pombe: JG580: h? cig2-Y136A-mNeonGreen:kanMX6 sid4-mRFP-kanMX6 ade6- leu1-32 ura4-D18	This manuscript	JG580
S. pombe: JG581: h? cig2-Y136A-mNeonGreen:kanMX6 sid4-mRFP-kanMX6 ade6- leu1-32 ura4-D18	This manuscript	JG581
S. pombe: JG582: h? cig2-Y136A,M168A,L172A,W175A-mNeonGreen:kanMX6 sid4-mRFP:kanMX6 ade6- leu1-32 ura4-D18	This manuscript	JG582
S. pombe: JG583: h? cig2-Y136A,M168A,L172A,W175A-mNeonGreen:kanMX6 sid4-mRFP:kanMX6 ade6- leu1-32 ura4-D18	This manuscript	JG583

**Recombinant DNA**		

leu1Δ::P.cdc13-cdc13-sfGFP-Tcdc13:hphMX6	Basu et al.^7^	ARC1300
leu1Δ::P.cdc13-cdc13-M235A,L239A,W242A-sfGFP-T.cdc13:hphMX6	Basu et al.^7^	ARC1301
leu1Δ::P.cdc13-cdc13-T.cdc13:hphMX6	Basu et al.^7^	ARC 1305
leu1Δ:cdc13P-cdc13-M235A,L239A,W242A-T.cdc13:hphMX6	Basu et al.^7^	ARC 1306
pFA6a_KanMX-tetO-pCyc1 (referred to as P.tet)	Zilio et al.^39^	Addgene 41023
pDM291-tetR-tup11Δ70	Zilio et al.^39^	Addgene 41027
pFA6a-GBP-mCherry:kanMX6	Chen et al.^40^	Addgene 89068
PCST3_lys1+:P.tet-cdc2-asM17-GBP-mcherry:natR	This manuscript	ARC1380
P.cig2-cig2-T.cig2-natMX6	This manuscript	ARC1381
P.cig2-cig2- M168A,L172A,W175A-T.cig2-natMX6	This manuscript	ARC1382
P.cig2-cig2-mNeonGreen-T.adh1-kanMX6	This manuscript	ARC1383
P.cig2-cig2-M168A,L172A,W175A-mNeonGreen-T.adh1:kanMX6	This manuscript	ARC1384
P.cig2-cig2-Y136A-mNeonGreen-T.adh1-kanMX6	This manuscript	JGp75
P.cig2-cig2-Y136A,M168A,L172A,W175A -mNeonGreen-T.adh1:kanMX6	This manuscript	JGp76

**Software and algorithms**		

FlowJo v10.7.2	FlowJo	https://www.flowjo.com/
Prism 9	GraphPad	https://www.graphpad.com/
MATLAB R2022b	Mathworks	https://www.mathworks.com/products/matlab.html
FIJI	Schindelin et al.^41^	https://imagej.net/ij/
Ilastik v1.4.0rc8	Berg et al.^42^	https://www.ilastik.org/
Perseus v1.6.14.0	Tyanova et al.^43^	https://maxquant.net/perseus/
MaxQuant v1.6.14	Cox et al.^44^	https://maxquant.net/maxquant/
Trackmate	Ershov et al.; Tinevez et al.^45^^,^^46^	https://imagej.net/plugins/trackmate/

### Resource availability

#### Lead contact

Further information and requests for resources and reagents should be directed to and will be fulfilled by the lead contact, Emma Roberts (emma.roberts@path.ox.ac.uk).

#### Materials availability

*S. pombe* strains and plasmids generated in this study are listed in the key resources table and available from the lead contact upon request.

#### Data and code availability

• The mass spectrometry proteomics data obtained in this study have been deposited to the ProteomeXchange Consortium^39^ via the PRIDE^40^ partner repository. The accession number for the data is PRIDE:PXD044732. All other data is available from the lead contact upon request.
• All original code has been deposited at GitHub and is publicly available via Zenodo. The DOI is listed in the key resources table.
• Any additional information required to reanalyse the data reported in the paper is available from the lead contact upon request.

### Experimental model and study participant details

#### Fission yeast strain construction and growth conditions

Fission yeast media and cell culture techniques are described previously.^41^ Cells were grown in Edinburgh minimal media (EMM) supplemented with adenine, leucine and/or uridine at 0.15 g/L where required, other than cells in Figure S4B, which were grown in rich YE (yeast extract) media supplemented with adenine, leucine, uridine and histidine at 0.15 g/L. Cells were grown at 32°C apart from when imaging Cig2-mNG in an asynchronous population (Figures 4C–4E; S4B), for which cells were grown at 25°C G1 arrests by nitrogen starvation (detailed below), were also performed at 25°C. Strains PN6033 and PN6034, which include the tetracycline-inducible *P.tet-cdc2-asM17-GBP-mCherry*, were grown in media supplemented with 10 μM 1-NmPP1 before the experiment to inhibit activity of any Cdc2-asM17-GBP-mCherry protein produced by potential leaky expression. Strain construction was performed by mating and subsequent random spore analysis, or by transformation^41^ and confirmed by colony PCR. The following plasmids were both gifts from Michael Nick Boddy and bought from Addgene: pFA6a-KanMX-tetO-pCyc1 (P.tet) (Addgene plasmid # 41023; http://n2t.net/addgene:41023; RRID:Addgene_41023); and pDM291-tetR-tup11Δ70 (Addgene plasmid # 41027; http://n2t.net/addgene:41027; RRID:Addgene_41027).^42^ pFA6a-GBP-mCherry-kanMX6 was a gift from Quanwen Jin (Addgene plasmid # 89068; http://n2t.net/addgene:89068; RRID:Addgene_89068).^43^ The strains used in this study and the related plasmids used to construct them are listed in the key resources table.

### Method details

#### Cell-cycle arrest and release

G1 arrest of cells was performed by taking an exponentially growing culture at a density of 2 x 10^6^ cells/mL and washing at least three times in media containing no ammonium chloride and no supplements. Cells were held in the nitrogen starvation for between 16 and 19 h at 25°C, before release by washing into EMM media which contained ammonium chloride with the relevant supplements. To repress the thiamine-repressible *nmt41* promoter, thiamine hydrochloride (Sigma) dissolved in water was added to a final concentration of 30 μM 1 h before release from the nitrogen arrest and to the media used to release the cells. To induce protein from the *P.tet* promoter described previously,^42^ anhydrotetracycline hydrochloride (Sigma) dissolved in DMSO was added to a final concentration of 1.25 μg/mL.

#### Fluorescence microscopy and image analysis

Fluorescence microscopy was performed with a Nikon Ti2 inverted microscope, equipped with a Perfect Focus System and Okolab environmental chamber, and a Prime sCMOS or BSI camera (Photometrics). Micro-Manager v2.0 software (Open-imaging) was used to control the microscope.^44^ Fluorescence excitation was carried out using a SpectraX LED light engine (Lumencor) fitted with the following standard filters: 395/25 for imaging DAPI/calcofluor, 470/24 for imaging sfGFP/eGFP; and 575/25 for imaging mRFP; with either a dual-edge ET-eGFP/mCherry dichroic beamsplitter (Chroma 59022bs), or a 409/493/573/652 nm BrightLine quad-edge dichroic beamsplitter (Semrock). The following emission filters were used: Semrock, 438/24 nm BrightLine single-band bandpass filter for imaging DAPI/calcofluor; Chroma, ET - EGFP single-band bandpass filter ET525_50m for imaging sfGFP/eGFP; and Semrock, 641/75 nm BrightLine single-band bandpass filter FF02_641_75 for imaging mCherry/mRFP. A 100× Plan Apochromat oil-immersion objective (NA 1.45) was used and microscopy performed at 25°C for still imaging, or 32°C for time-lapse.

Time-lapse imaging was performed on agar pads, made by dissolving agarose to a final concentration of 2% w/v in EMM media supplemented with leucine, adenine and thiamine. The solution was used to fill a gene frame (Thermo Fisher) and allowed to solidify. Cells were mounted in their growth media on the agar pads, and imaged for 4.5 h, starting 1.5 h after release from the G1 arrest. FIJI software was used to measure pixel intensity, adjust brightness and contrast, apply a pixel range to images, and render maximum projection images.^45^ Where images are shown, the same pixel range has been applied to all images of the same channel within a figure panel, unless otherwise stated.

For analysis of still imaging, cells were segmented using Ilastik.^46^ For the whole-cell intensity measurements of Cig2-mNG in an asynchronous population (Figure 4E), background value of autofluorescence was subtracted from the measurements. To find the background value of autofluorescence, the mean cellular fluorescence intensity was measured of a population of cells with the same genetic background but without Cig2-mNG, and the median of this population was taken as the value of autofluorescence.

For analysis of time-lapse images, for faster segmentation, cells were segmented and labeled using a custom script in MATLAB 9.13.0 R2022b (Mathworks). To identify edges of cells more clearly, cells were segmented on images 1 μm below the focal plane. First, cells were segmented using the “imbinarize” function with an adaptive threshold algorithm. Any regions of the out-of-cell background were removed using an intensity threshold as cells contained higher intensity values. Any gaps formed inside cells were filled using the “imfill” function. The binary masks of cells were eroded to facilitate the detection of cell boundaries. A watershed algorithm was used using the “watershed” function to identify cells and their boundaries. The resulting masks were dilated to restore their normal size and the watershed algorithm was used again to check if cell boundaries were accurately detected. Holes in the binary masks were filled using the “imfill” function. Any subsequent out-of-cell artifacts were removed using size and intensity thresholds. The masks of cells were labeled with numbers using the “labelmatrix” function for tracking analysis, with manual corrections made as necessary. Labeled cells were then tracked using the Lineage Mapper plugin in FIJI.^47^ Whole-cell intensity measurements were performed by taking the mean fluorescence intensity in the cell mask.

Spot detection to identify Cdc13 and Cig2 localisation to the SPB was performed using the trackmate v7.10.0 FIJI plugin.^48^^,^^49^ A DoG detector was used for all experiments with the estimated spot diameter set to 0.35 μm, and the following parameters selected: pre-process with median filter; sub-pixel localisation. The parameters used to filter spots were determined empirically for each different experiment, with the same settings being used between all strains and all replicates of any given experiment. Due to Sid4-mRFP signal being close to background in the time-lapse, Sid4-mRFP spots were not detected in all frames of the timelapse; to account for this the spot detection workflow outlined in Figure S1 was adapted. Cells were counted as having Cig2-mNG at the SPB if they had either a Cig2-mNG spot that intersected with a Sid4-mRFP spot in any given frame of the timelapse, or a Cig2-mNG spot in two out of three consecutive frames with no detected Sid4-mRFP spot.

#### Flow cytometry and cell cycle progression analysis

DNA content analysis was performed using flow cytometry. Cells were fixed in 70% ice-cold ethanol, before being washed and resuspended in 50 mM sodium citrate. Samples were incubated with 0.1 mg/mL RNase A at 37°C for at least 3 h. DNA was stained with Sytox Green (1 μM, Invitrogen). 100,000 events were acquired on either an LSRII or BD LSRFortessa, using FACS Diva software. DNA content is displayed on a linear scale after gating for single cells in FlowJo v10.7.2 (Treestar Inc). Analysis of the percentage of cells with 1C DNA content in Figure 4B was performed by gating around the 1C peak in the histograms represented in Figure 4A.

Binucleation analyses and detection of aberrant divisions were performed by heat-fixing cells on a microscope slide at 70°C before staining with 4′,6-diamidino-2-phenylindole (DAPI) (SlowFade Diamond Antifade Mountant with DAPI, Invitrogen) and Calcofluor (Sigma). Cells that displayed the ‘*cut*’ phenotype (a septum intersecting an undivided nucleus, or two foci of DAPI staining very close to a septum) were included in the ‘aberrant’ category, as were cells that had a single nucleus on one side of a septum, or multiple septa.

#### Protein extraction and phosphoproteomics

Protein extracts were prepared by addition of ice-cold 100% w/v trichloroacetic acid (TCA) to a final volume of 10% to a cell culture. Cells were kept on ice for at least 30 min before being washed in ice-cold acetone. Dry pellets were stored at −80°C, before being washed and resuspended in lysis buffer (8 M urea, 50 mM ammonium bicarbonate, 5 mM ethylenediamine tetraacetic acid (EDTA), 1 mM phenylmethylsufonyl fluoride (PMSF), 1X protease inhibitor cocktail set III (EDTA free, CalBiochem), and 1X PhosSTOP phosphatase inhibitor cocktail (Roche)). Cells were lysed by two rounds of beating with glass beads at 4°C using a FastPrep120 at 5.5 m/s for 45 s, followed by one round of beating at 6.5 m/s for 40 s, with the samples placed on ice for 2 min between rounds of beating. The supernatant was collected after cell debris was pelleted at 13,000 rpm in a table-top microcentrifuge at 4°C.

For phosphoproteomic analysis, lysates (200 μg) were digested using SP3 beads according to manufacturer instructions using Trypsin and LysC. Following digestions samples were labeled with TMT10plex Isobaric Label Reagents kit (Thermo Fisher) as previously described,^50^ with the exception that peptides were desalted using a desalting column (89852, Thermo Fisher). Samples were normalised using label amounts as previously described^51^ and subjected to sequential TiO2 and Fe-NTA phosphopeptide enrichment protocols according to manufacturer instructions. Resulting phosphopeptides and whole cell lysates (flow through) were fractionated using Pierce High pH Reverse-Phase Peptide Fractionation kit (Thermo Fisher). Samples were dried and reconstituted in 0.1% trifluoroacetic acid (TFA). Peptides were loaded onto trap column and separated using Easy Spray 50cm (Ultimate3000) using 180 min gradient (mobile phase A 0.1% TFA – 95%, 5% DMSO, mobile phase B −75 %ACN, 5% DMSO, 5% water). For the gradient buffer B concentration was 2% - 0min, 8%-5.5min, 40%- 153min, 95%-155min, 2%-165min. Data were acquired using Orbitrap Eclipse using SPS-MS3 method with the following settings: for MS1 Orbitrap resolution was set to 120000, AGC target to standard and automatic injection time, for MS2 Orbitrap resolution was set at 30000 with fragmentation CID 35, and standard AGC target and injection time, and for MS3 Orbitrap resolution was set to 50000, HCD fragmentation was set at 65 and AGC target was set to 200% with 60ms maximum injection time.

#### Phosphoproteomic data analysis

The dataset was searched on MaxQuant^52^ v1.6.14 against a *Schizosaccharomyces pombe* proteome FASTA file extracted from UniProt,^53^ amended to include common contaminants and the introduced exogenous Cdc2-asM17-GBP-mCherry construct. The default MaxQuant parameters were used with Phospho(STY) added as a variable modification. Subsequent analysis was performed in Perseus v1.6.14.0.^54^ Different multiplicities of the same site were treated as separate phosphorylation events. The data was filtered to remove contaminant and reverse hits, and to retain only sites that appeared in all time-points of the time-course and had a localisation probability of at least 0.7. All phosphorylation events were normalised to the median value of their time-point to account for potential mixing errors. All time-points of all phosphorylation events were then normalised to their respective T0. Sites were further normalised to their respective T0-normalised protein change (sites without proteome data for all time points were excluded). In all line graphs where phosphoproteomic data is presented, the data is presented in this format (protein-normalised, T0-normalised phosphorylation) and this is referred to as the normalised phosphorylation.

To calculate the max phosphorylation ratio of a given phosphorylation event, the maximum normalised phosphorylation reached in the time-course was identified for both strains. The ratio was calculated using: SPB-tethered max phosphorylation/control max phosphorylation. Substrate localisation information was compiled from published literature in our previous study.^7^

### Quantification and statistical analysis

Statistical tests performed are detailed in the figure legends and were performed using GraphPad Prism 9. Normality was checked by the D’Agostino and Pearson normality test, and Mann-Whitney rank comparison was used to compare conditions that were not normally distributed.

## Supplemental information

Document S1. Figures S1–S4

Table S1. Condensed phosphoproteomic dataset, related to Figures 3 and S3

Document S2. Article plus supplemental information
